# Plasma Nesfatin-1 Is Not Affected by Long-Term Food Restriction and Does Not Predict Rematuration among Iteroparous Female Rainbow Trout (*Oncorhynchus mykiss*)

**DOI:** 10.1371/journal.pone.0085700

**Published:** 2014-01-09

**Authors:** Lucius K. Caldwell, Andrew L. Pierce, Larry G. Riley, Christine A. Duncan, James J. Nagler

**Affiliations:** 1 Department of Biological Sciences & Center for Reproductive Biology, University of Idaho, Moscow, Idaho, United States of America; 2 Columbia River Inter-Tribal Fish Commission, Portland, Oregon, United States of America; 3 Department of Biology, Fresno State University, Fresno, California, United States of America; University of Hong Kong, Hong Kong

## Abstract

The metabolic peptide hormone nesfatin-1 has been linked to the reproductive axis in fishes. The purpose of this study was to determine how energy availability after spawning affects plasma levels of nesfatin-1, the metabolic peptide hormone ghrelin, and sex steroid hormones in rematuring female rainbow trout (*Oncorhynchus mykiss*). To limit reproductive maturation, a group of female trout was food-restricted after spawning and compared with a control group that was fed a standard broodstock ration. The experiment was conducted twice, once using two-year-old trout (second-time spawners) and once using three-year-old trout (third-time spawners). During monthly sampling, blood was collected from all fish, and a subset of fish from each treatment was sacrificed for pituitaries. Pituitary follicle-stimulating hormone-beta (*fsh-β*) mRNA expression was analyzed with q-RT-PCR; plasma hormone levels were quantified by radioimmunoassay (17*β*-estradiol and ghrelin) and enzyme-linked immunosorbent assay (11-keto-testosterone and nesfatin-1). Although plasma nesfatin-1 levels increased significantly in the months immediately after spawning within both feeding treatments, plasma nesfatin-1 did not differ significantly between the two treatments at any point. Similarly, plasma ghrelin levels did not differ significantly between the two treatments at any point. Food restriction arrested ovarian development by 15–20 weeks after spawning, shown by significantly lower plasma E2 levels among restricted-ration fish. Pituitary *fsh-β* mRNA levels were higher among control-ration fish than restricted-ration fish starting at 20 weeks, but did not differ significantly between treatment groups until 30 weeks after spawning. Within both treatment groups, plasma 11-KT was elevated immediately after spawning and rapidly decreased to and persisted at low levels; starting between 20 and 25 weeks after spawning, plasma 11-KT was higher among control-ration fish than restricted-ration fish. The results from these experiments do not provide support for plasma nesfatin-1 as a signal for the initiation of reproductive development in rematuring female rainbow trout.

## Introduction

The reproductive endocrine axis–comprising the brain, pituitary, and gonads (BPG)–has been characterized for some fishes [1], [2], [3] including salmonids [4], and the basic mechanism by which the BPG axis secretes hormones to regulate initial maturation (puberty) in salmonid species are well understood [5]. However, there is a dearth of information concerning endocrine regulation and coordination of gonadal recrudescence (rematuration). While it is reasonable to suspect that puberty and rematuration are regulated similarly, this hypothesis has mostly gone untested. Furthermore, although the role of energetics in puberty is well described in many animals [6], [7], [8] including fishes [9], [10], [11], how metabolic and nutritional status regulate successive reproductive efforts has largely been overlooked.

In mammals, nesfatin-1 is an 82-amino acid hormone cleaved from the nucleobindin-2 (*nucb2*) gene product that is secreted primarily from the hypothalamus and adipose tissue, and that has both anorexigenic and growth inhibiting effects [12]. In rats, short-term (24-hour) fasting leads to a decrease in circulating levels of nesfatin-1, which return to normal levels upon refeeding [13]. In addition to behavioral and metabolic effects, nesfatin-1 appears to inform the reproductive endocrine axis regarding nutritional status [14]: in pigs, nesfatin-1 injections elevated plasma luteinizing hormone (LH) levels [15]. In goldfish (*Carassius auratus*), *nucb2* mRNA is widely expressed in the brain and peripheral tissues, with the highest expression levels found in the liver and pituitary [16], nesfatin-1 injections reduce feeding [16], and nesfatin-1 suppresses the BPG axis at all three tiers [17]. Taken together, these results suggest that circulating nesfatin-1 may act to integrate metabolic or feeding status with reproductive development.

The metabolic peptide hormone ghrelin is synthesized and secreted from the stomach of vertebrates during an energy deficit [18]. In concert with other circulating endocrine factors, ghrelin coordinates the metabolic response to fasting and has behavioral effects such as stimulating appetite in salmonid fishes [19], [20]. Ghrelin and nesfatin-1 colocalize in stomach and hypothalamus of goldfish [21]. In unfed goldfish, i.c.v. nesfatin-1 injection suppressed preproghrelin and ghrelin receptor mRNA expression in forebrain, suppressed ghrelin and NUCB2 mRNA expression in hypothalamus, and suppressed NUCB2 mRNA expression in forebrain [21]. Along with leptin and other regulatory metabolic hormones, ghrelin acts at multiple levels of the BPG axis to inhibit reproduction, *e.g.*, by suppressing pituitary LH and testicular androgen secretion [22]. Ghrelin acts directly on zebrafish follicles to inhibit both basal and MIH-induced germinal vesicle breakdown [23]. Ghrelin abnormalities have been implicated in a variety of human reproductive disorders [24], [25]. Thus, ghrelin may act as a peripheral metabolic signal that negatively regulates reproductive development under conditions of nutritional deficit among iteroparous fishes such as rainbow trout [7], [26], [27].

Among salmonid fishes, the onset of puberty occurs up to a year in advance of spawning [28], [29]. The current working hypothesis states that puberty in salmonids is ultimately regulated by energy availability [7], [26]. When energy is deficient, some peripheral metabolic hormone inhibits or arrests reproductive development [23], [27]. When energy is sufficient, some peripheral metabolic hormone stimulates or permits the reproductive axis to proceed with reproductive development [17], [30]. Upon receiving the stimulatory or permissive signal from such a metabolic cue, maturation is initiated in the brain by gonadotropin releasing hormones (GnRHs). GnRHs originate primarily in the hypothalamus [31] and act hierarchically to stimulate pituitary release of the gonadotropin hormones (GtHs), follicle stimulating hormone (FSH) and luteinizing hormone (LH) [32]. In female fishes, the GtHs stimulate growth and development of the ovaries [33], and also activate steroidogenic pathways culminating in secretion of both the estrogenic sex steroid 17*β*-estradiol (E2) and the androgenic sex steroid 11-keto-testosterone (11-KT) [34], [35]. Increasing levels of circulating E2 both initiate and signal ovarian maturation [36], [37]. Recent work suggests a role for 11-KT in early oocyte growth among various species of teleost fishes [38], [39], [40]; *in vitro* evidence suggests such a role for 11-KT in salmonids [41].

The purpose of this study was to determine how energy availability after spawning affects plasma levels of nesfatin-1, ghrelin, and sex steroid hormones in rematuring female rainbow trout (*Oncorhynchus mykiss*). We hypothesized that by restricting food availability in a group of female trout, these fish would become energy deficient and thus arrest reproductive development [42] as individuals adopted a non-consecutive year or “skip-spawner” life history that has been described in fishes [43], [44]. We also hypothesized that this energy deficit would be initially reflected by increased plasma ghrelin and decreased plasma nesfatin-1 [43], [44]. We further hypothesized that this skip-spawner life history decision would be detectible first by reduced pituitary levels of *fsh-β* mRNA, then by reduced circulating levels of 11-KT, and finally by reduced circulating levels of E2.

## Materials and Methods

### 1. Ethics Statement

Experiments were approved by and conducted under approved protocols in accordance with the principles and procedures of the Institutional Animal Care and Use Committee, University of Idaho (Permit Number 2012-130). Fish were anesthetized with tricaine methanesulfonate (Finquel MS-222, Argent Laboratories, Redmond, WA) prior to handling.

### 2. Animals

Post-spawned female rainbow trout (*Oncorhynchus mykiss*) were purchased from Troutlodge (Sumner, WA) and transported to the University of Idaho (Moscow, ID). Fish had been manually strip-spawned 2 d prior to transport, and were fasted for one month (in the case of two-year-old trout) or two months (in the case of three-year-old trout) prior to spawning. Fish were held in 1,130 L tanks, in a recirculating system (flow rate 14 L min^−1^ per tank, temperature 12 to 15°C following a seasonal profile).

### 3. Experiments

Experiments were conducted as previously described [42]. Briefly, 150 two-year-old or three-year-old fish were started at week zero on a control ration (0.5% fish mass per day), or a restricted ration (0.1% fish mass per day). Fish were fed a commercial trout broodstock diet (6.4 mm pellets, Rangen, Inc., Buhl, ID), and were sampled every 5 weeks (two-year-old fish) or every 4 weeks (three-year-old fish). During sampling, blood was drawn from all fish, and a subsample of fish from each treatment group was lethally sampled for tissue collection (causing *n* to decrease as weeks progressed; see figure legends for relevant *n* for each analysis). PIT tags individually identified fish.

### 4. RNA Extractions & cDNA Synthesis

Pituitary samples were homogenized in 1.0 mL ice cold TRIzol (Invitrogen, Life Technologies, Carlsbad, CA), and RNA was isolated following the TRIzol protocol, using three chloroform:isoamyl alcohol extractions and three 70% ethanol washes. Resuspended nucleic acid fractions were treated with DNase (TURBO DNA-free, Ambion, Life Technologies, Carlsbad, CA). RNA purity was assessed by spectrophotometric absorbance (NanoDrop ND-1000, Thermo Fisher), and RNA concentration was measured using the RiboGreen RNA assay kit (Invitrogen) with a fluorometer. 1 µg total RNA was reversed transcribed with the SuperScript III First-Strand Synthesis Kit (Invitrogen) using random hexamer primers. cDNA was diluted 1∶5 in 1× Tris-EDTA.

### 5. q-RT-PCR

A quantitative real-time reverse-transcriptase PCR (q-RT-PCR) primer set (Table 1) was designed using ABI Primer Express 3.0 software (Life Technologies, Carlsbad, CA) to amplify a 60 bp fragment of the annotated *O. mykiss fsh-β* sequence (NM_001124586). Specificity was confirmed by bioinformatic analysis, agarose gel electrophoresis of PCR products, and melting curve analysis of PCR products. To carry out the q-RT-PCR, sample cDNA was amplified in 96-well optical reaction plates (Invitrogen) containing 20 µL PCR reactions made up of 2 µL cDNA, 10 µL *Power* SYBR Green PCR Master Mix (Life Technologies), 6 µL H_2_O, and 2 µL of a mix of forward and reverse primers at 2 pM each, in an Applied Biosystems ABI 7900 HT real-time PCR system (Life Technologies) (2 min @ 50°C; 10 min @ 95°C; 40 cycles of 15 sec @ 95°C and 1 min @ 60°C). Copy numbers in samples were quantified using standard curves of PCR amplicons. Positive and negative controls were included on each plate. Three technical replicate PCRs were completed for each sample. The mean of the mRNA copy number for the three replicate PCRs is the value reported. q-RT-PCR results were log_2_-transformed prior to statistical analysis.

**Table 1:** Primer sequence data for q-RT-PCRs.

Gene	Accession Number	Direction	Sequence	Product Size
*fsh-β*	AB050835	Fwd	AGAGCTGCGATTGCATCAAA	61 bp
		Rev	GCCATGCTTATGCGATCACA	

### 6. 17β-Estradiol Radioimmunoassay (RIA)

#### 6.1. Solvent extraction

Plasma samples were extracted with methyl tert-butyl ether (MTBE) (Fisher Scientific, Hampton, NH) by combining 100 µL plasma with 4.0 mL MTBE and vortexing for 1 min [45]. Samples were incubated at room temperature for 7 min to allow phase separation to occur and then the aqueous phase was frozen. The solvent fraction was decanted, equilibrated 10 min at room temperature and incubated at 55°C until all solvent had volatilized (approximately 2 hour). A second extraction of the remaining aqueous fraction from each plasma sample was performed, using 3.0 mL MTBE, and pooled with the first extract. Dried extracts were resuspended in 250 µL E2 zero calibrator solution from the E2 RIA kit (Coat-A-Count, Siemens, Munich, Germany). Average extraction efficiency was 83%, as determined by RIA values for extracted versus unextracted assay standards included with the RIA kit.

#### 6.2. RIA

Resuspended plasma extracts were analyzed in duplicate for E2 concentration using an antibody-coated tube E2 radioimmunoassay (RIA) kit (Coat-A-Count, Siemens, Munich, Germany), per the manufacturer’s instructions. Sensitivity for the assay is reported to be 8 pg mL^−1^. Positive and negative controls were included in all assays.

### 7. 11-keto Testosterone Enzyme-linked Immunosorbent Assay (EIA)

#### 7.1. Solvent extraction

Plasma samples were extracted with anhydrous diethyl ether (JT Baker, Avantor Performance Materials, Inc.; Center Valley, PA, USA) by combining 100 µL plasma with 2.0 mL diethyl ether and vortexing for 1 min [46]. Samples were incubated at room temperature for 7 min to allow phase separation to occur, and then the aqueous phase was frozen. The solvent fraction was decanted. A second extraction of the remaining aqueous fraction from each plasma sample was then performed, using 2.0 mL diethyl ether, and pooled with the first extract. Diethyl ether extracts were dried down in a 49°C water bath under a gentle stream of N_2_ directed *via* a nitrogen evaporator manifold (N-EVAP 112; Organomation Associates, Inc; Berlin, MA). Dried extracts were resuspended in 1000 µL EIA buffer from the 11-KT EIA kit (described below). Extraction efficiency ranged from 90–102%, as determined by RIA values for extracted versus unextracted assay standards included with the RIA kit.

#### 7.2. EIA

Resuspended plasma extracts were analyzed in duplicate for 11-KT concentration using an antibody-coated 96-well plate based enzyme-linked immunosorbent assay (EIA) kit (Cayman Chemical Company; Ann Arbor, MI). Sensitivity for the assay is reported to be 1 pg mL^−1^. Positive and negative controls were included in all assays.

### 8. Ghrelin RIA

Plasma ghrelin concentrations were measured in duplicate using a ghrelin radioimmunoassay established and validated for use in fish by Riley et al. [47], with the following minor variations. One hundred µL of rat ghrelin standards and plasma samples were incubated with 200 µL anti-rat ghrelin (from H. Hosoda) at a dilution of 1∶750,000. The anti-rat ghrelin (a.a. 1–11) recognizes the octanoylated epitope (biologically active region) of ghrelin [48] and detects only the biologically active forms of ghrelin (ghrelin-C8 and ghrelin-C10). After incubation at 4°C overnight, 100 µL of ^125^I-human ghrelin (Millipore, St. Charles, MO) was added before an additional overnight incubation at 4°C. Finally, 100 µL anti-rabbit IgG goat serum at 1∶35 (with 10% polyethylene glycol) was added, incubated for 2 hour at room temperature, and then centrifuged at 3200×g for 60 min to separate free and bound tracers. Radioactivity of aspirated pellet was then quantified using a gamma counter (Packard, Palo Alto, CA). Intra- and inter-assay CV’s were 6.2 and 9.8%, respectively. Positive and negative controls were included in all assays.

### 9. Nesfatin-1 EIA

#### 9.1. Parallelism of trout plasma dilution

**Figure 1:**
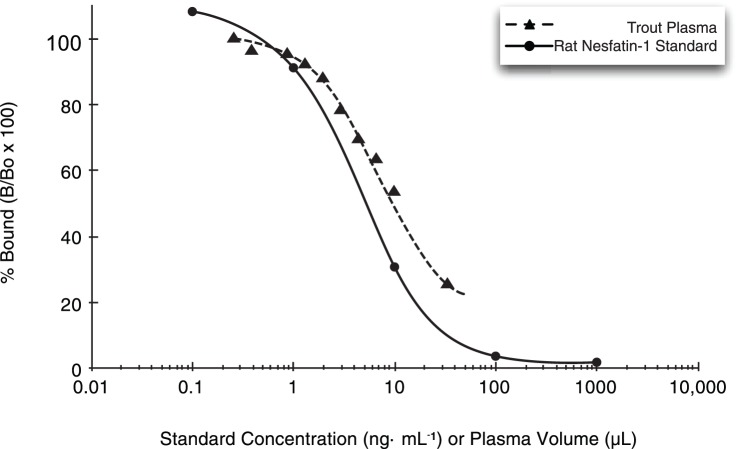
Parallel displacement of rat nesfatin-1 by a serial dilution of trout plasma. Displacement curve showing parallelism of the rat nesfatin-1 standard curve (circle points, solid line) and a dilution series of female rainbow trout plasma displacing rat-nesfatin-1 (triangle points, dashed line) in the nesfatin-1 EIA. Horizontal axis depicts volume of trout plasma (µL) used in the assay or concentration of rat-nesfatin-1 standard (ng⋅mL^−1^).

A rat nesfatin-1 EIA (EK 003–22; Phoenix Pharmaceuticals) was used for measuring nesfatin-1 immunoreactivity (nesfatin-1) in rainbow trout plasma. Although this kit has been previously validated for use in fish (goldfish [16]), the following procedure was conducted to establish its use in rainbow trout. A representative sample of female trout plasma was serially diluted 1.5 fold in 1× nesfatin-1 EIA buffer and measured using the nesfatin-1 EIA; displacement of label by standard and by trout plasma were then compared (Fig. 1). A four-parameter logarithmic regression line was fit to the relationship between displacement and plasma volume. Displacement of rat nesfatin-1 by trout plasma was parallel to the standard curve, indicating the presence of an immunologically similar protein in trout plasma. Displacement was linear over the range 1.3 µL– 9.9 µL trout plasma (R^2^ = 0.99).

#### 9.2. Measuring nesfatin-1 levels in trout plasma

Rainbow trout plasma samples were assayed for nesfatin-1 by diluting them 1∶10 in 1× EIA buffer to bring values within the linear range of the kit, as described above. Samples were run in duplicate, and had intra-sample CV values <10%. Positive control samples were all within the range specified in literature accompanying the kit, and negative controls were included for all assays.

### 10. Data Analysis

Only data from fish that survived until being terminally sampled were included in statistical analyses. Systematic tank differences were not detected within treatment for any variable (ANOVA, *p*>0.05). Therefore, tank replicates were pooled and analyzed together. For normality and homoscedasticity requirements of ANOVA and post-hoc tests, plasma hormone data were log-transformed prior to analysis. Two-way ANOVA was used to detect main and interaction effects (time, treatment, time*treatment). When ANOVA indicated a significant time effect, Tukey-Kramer Honestly Significant Difference (Tukey-Kramer HSD) tests were used to compare values at all time points within a given treatment. Within each time point, two-tailed t-tests were used to detect treatment differences. Statistical analyses were performed with JMP (Version 9, SAS Institute Inc., Cary, NC). Differences are reported as significant when *p*<0.05.

## Results

**Figure 2:**
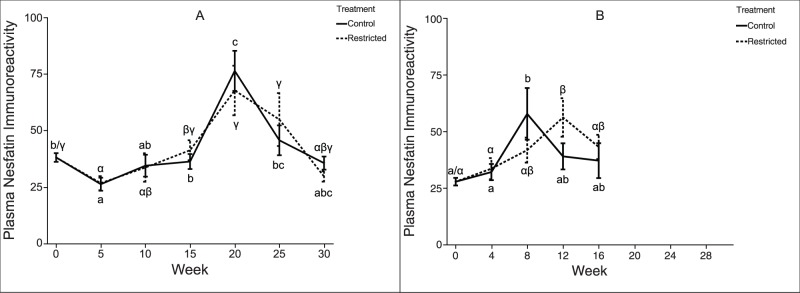
Plasma nesfatin-1 concentrations in female rainbow trout recovering from spawning. Mean (±SEM) plasma nesfatin-1 concentration over time in female rainbow trout (A: two-year-old; B: three-year-old) fed a control-ration or restricted-ration. When comparing treatment groups, mean values differ significantly (two-tailed *t*-test, *p*<0.05) at time-points marked “*” and marginally (two-tailed *t*-test, *p*<0.1) at time points marked “^‡^”. Within each figure, time-points sharing the same letter are not significantly different (Tukey’s HSD, *p*≥0.05). Latin letters (a, b, c, …) refer to control treatment fish; Greek letters (α, β, γ, …) refer to restricted ration fish. Figure A: Week 0, *n* = 42 per treatment; Week 5, *n* = 36 per treatment; Week 10, *n* = 30 per treatment; Week 15, *n* = 24 per treatment; Week 20, *n* = 18 per treatment; Week 25, *n* = 12 per treatment; Week 30, *n* = 6 per treatment. Figure B: Week 0, *n* = 25 per treatment; Week 4, *n* = 20 per treatment; Week 8, *n* = 15 per treatment; Week 12, *n* = 10 per treatment; Week 16, *n* = 5 per treatment.

**Figure 3:**
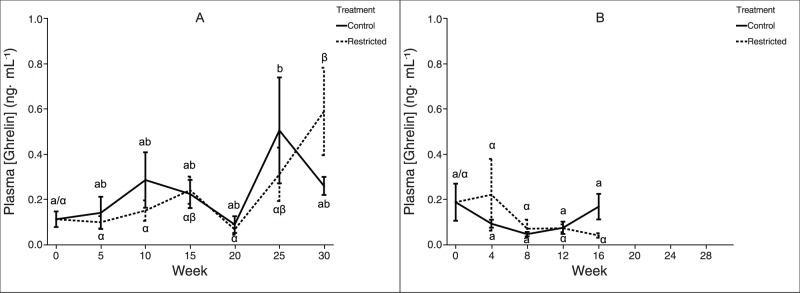
Plasma ghrelin concentrations in female rainbow trout recovering from spawning. Mean (±SEM) plasma acylated ghrelin levels over time in female rainbow trout (A: two-year-old; B: three-year-old) fed a control-ration or restricted-ration. See Figure 2 for an explanation of what each symbol signifies. Figure A: *n* = 6 per treatment. Figure B: Week *n* = 5 per treatment.

As described previously [42], the feeding regime used here affected metrics of growth, metabolism, and reproduction in both two- and three-year-old rematuring female rainbow trout. However, in both experiments there was no significant difference in plasma nesfatin-1 immunoreactivity between the treatment groups at any time point (Fig. 2). Within each experiment, a two-way ANOVA determined that “week” was a significant effect, but neither “treatment” nor the “treatment*week” interaction were significant effects. Plasma nesfatin-1 immunoreactivity covaried significantly in the two treatment groups (ANCOVA, p<0.0001), suggesting that plasma nesfatin-1 was not affected by treatment. While the feeding regimes used here did not yield differences in trout plasma nesfatin-1 immunoreactivity between treatments, plasma nesfatin-1 immunoreactivity did change over time both within each treatment individually and when the feeding treatments were pooled and considered together. In both two-year-old and three-year-old trout, there was a marked increase over time in plasma nesfatin-1 immunoreactivity, before levels declined toward the end of the experiment. Similarly, no difference in plasma levels of acylated-ghrelin (ghrelin) was detected either among time points within treatment groups for a given age class, or between treatment groups at any time-point for a given age class (Fig. 3).

**Figure 4:**
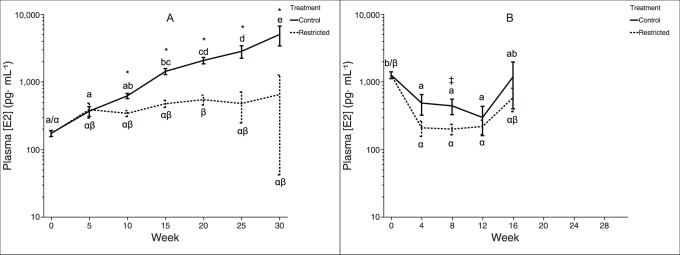
Plasma estrogen concentrations in female rainbow trout recovering from spawning. Mean (±SEM) plasma 17β-estradiol concentration over time in female rainbow trout (A: two-year-old; B: three-year-old) fed a control-ration or restricted-ration. See figure 2 for an explanation of what each symbol signifies. Figure A: Week 0, *n* = 42 per treatment; Week 5, *n* = 36 per treatment; Week 10, *n* = 30 per treatment; Week 15, *n* = 24 per treatment; Week 20, *n* = 18 per treatment; Week 25, *n* = 12 per treatment; Week 30, *n* = 6 per treatment. Figure B: Week 0, *n* = 25 per treatment; Week 4, *n* = 20 per treatment; Week 8, *n* = 15 per treatment; Week 12, *n* = 10 per treatment; Week 16, *n* = 5 per treatment.

The feeding treatments produced a significant difference in reproductive development during this period of initial ovarian recrudescence, as indicated by plasma E2 levels (Fig. 4): among two-year-old trout fed the control-ration, plasma E2 concentration was elevated compared to restricted-ration fish by week ten and continued to increase dramatically over the course of the experiment. Plasma E2 levels showed a similar but non-significant elevation in control versus restricted-ration fish among three-year-old trout starting at week eight. Interestingly, at week zero, three-year-old trout exhibited plasma levels of E2 that were an order of magnitude greater than those observed among two-year-old trout, before decreasing significantly as the experiment progressed.

**Figure 5:**
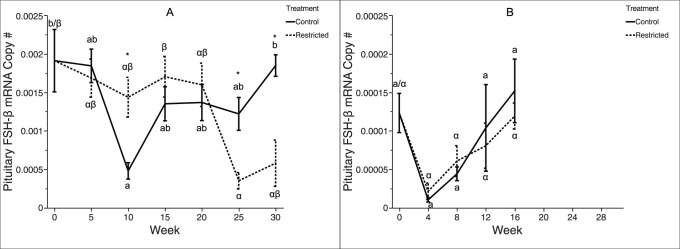
Pituitary *fsh-β* mRNA expression in female rainbow trout recovering from spawning. Mean (±SEM) normalized ratio of pituitary *fsh-β* to *β-actin* mRNA levels over time in female rainbow trout (A: two-year-old; B: three-year-old) fed a control-ration or restricted-ration. See Figure 2 for an explanation of what each symbol signifies. Figure A: *n* = 6 per treatment at all time points. Figure B: *n* = 5 per treatment at all time points.

Among two-year-old trout, pituitary *fsh-β* mRNA expression decreased from weeks 0–25 within restricted-ration fish, while *fsh-β* expression increased from weeks 10–30 within control-ration fish (Fig. 5). This trend led to a significant difference between the treatment groups: restricted-ration fish exhibited higher pituitary *fsh-β* mRNA levels shortly after spawning, at week 10, and control-ration fish exhibited higher pituitary *fsh-β* mRNA levels later, at weeks 25–30. Among three-year-old trout, pituitary *fsh-β* mRNA expression did not significantly change over time within either treatment group, and pituitary *fsh-β* mRNA expression levels did not significantly differ between the two groups at any time point.

**Figure 6:**
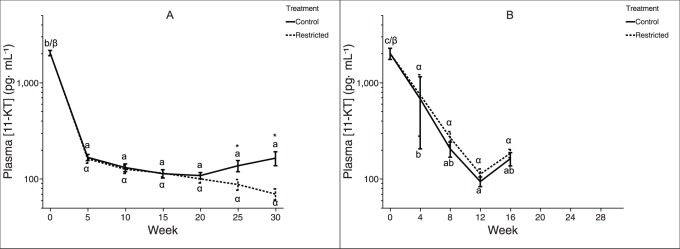
Plasma androgen concentrations in female rainbow trout recovering from spawning. Mean (±SEM) plasma 11-keto testosterone concentration over time in female rainbow trout (A: two-year-old; B: three-year-old) fed a control-ration or restricted-ration. See Figure 2 for an explanation of what each symbol signifies. Figure A: Week 0, *n* = 42 per treatment; Week 5, *n* = 36 per treatment; Week 10, *n* = 30 per treatment; Week 15, *n* = 24 per treatment; Week 20, *n* = 18 per treatment; Week 25, *n* = 12 per treatment; Week 30, *n* = 6 per treatment. Figure B: Week 0, *n* = 25 per treatment; Week 4, *n* = 20 per treatment; Week 8, *n* = 15 per treatment; Week 12, *n* = 10 per treatment; Week 16, *n* = 5 per treatment.

Plasma concentration of 11-KT (Fig. 6) showed a similar trend within both year classes: plasma 11-KT levels were elevated at week zero before precipitously declining and remaining low through the remainder of the experiment. Among two-year-old trout, plasma 11-KT diverged at week 25, with control-ration fish exhibiting significantly elevated plasma 11-KT levels compared to restricted-ration fish; the experiment with three-year-old trout was presumably not long enough to capture this effect, as no difference was detected.

## Discussion

In our previous report [42], we showed that the control-ration described in the current study was sufficient to maintain positive growth rates, while the restricted-ration was sufficient to maintain near-zero or negative growth rates. Our current results provide evidence that plasma nesfatin-1 levels are not affected by this long-term feeding restriction in post-spawned female rainbow trout; no difference in plasma nesfatin-1 levels was detected between the control-ration and restricted-ration fish at any time point in two-year-old or three-year-old trout. Additionally, circulating nesfatin-1 levels observed during the months after spawning do not predict rematuration in rainbow trout: restricted-ration fish failed to undergo initial sexual maturation, but nonetheless exhibited plasma nesfatin-1 levels that were similar to control-ration fish. While i.p. injections of nesfatin-1 have been shown to inhibit the reproductive endocrine axis in fish [17], the observed inhibitory effect may be due to relatively high (*i.e.*, pharmacological) dosage, and thus not accurately reflect the native (*i.e.*, physiological) role of circulating nesfatin-1. Alternatively, nesfatin-1 may act differently in fish with group-synchronous ovary development (rainbow trout) compared to fish with asynchronous ovary development (goldfish). Plasma nesfatin-1 levels did exhibit a pattern suggesting nesfatin-1 may be regulated seasonally in rainbow trout, with a peak occurring near the summer solstice (Fig. 2A). Further studies are required to determine the significance of seasonal changes in trout plasma nesfatin-1 levels.

Similarly, within both age classes, plasma ghrelin levels did not differ significantly between treatment groups at any time point. Among two-year-old trout fed the restricted-ration, plasma ghrelin did significantly increase between week 20 and week 30. However, plasma ghrelin levels also exhibited a transient spike among two-year-old trout fed the control-ration around week 25. Among three-year-old trout, the later increase in plasma ghrelin (between week 20 and week 30) was not observed; this is likely the result of the experiment using three-year-old trout not running long enough. Taken together, these findings do not support a role for ghrelin as an indicator of long-term nutritional status in rainbow trout. Although these data do not support our original hypothesis, this contradiction with previous results in other systems may be partially explained by the observation that ghrelin physiology differs substantially between fishes and mammals [49], [50]. For instance, in rainbow trout, ghrelin administration does not increase feed intake, and plasma ghrelin levels are reduced during fasting [20], [51]. Also in rainbow trout, *in vitro* ghrelin treatment of gastrointestinal tissue does not affect GI contractility [52]. In channel catfish, neither plasma ghrelin nor stomach *ghrl* mRNA expression changes with feeding status [53]. In two species of sturgeon [54], and in grass carp [55], ghrelin appears to play a principally developmental role. It appears that more work needs to be done to clarify the disparate roles of ghrelin among non-mammalian vertebrates. However, our results do not support regulation of plasma ghrelin levels by either recovery from spawning or nutritional status in post-spawned rainbow trout.

The experimental design used in this study affected nutrition sufficiently to arrest reproductive development among two-year-old trout, as evidenced by a divergence in plasma E2, GSI [42], plasma 11-KT and pituitary *fsh-β* mRNA expression. This suggests that the nutritional limitation imposed upon the restricted-ration fish sufficed to induce a physiological trade-off between investment in continued survival and growth versus investment in reproductive development. While the experiment using three-year-old trout did not continue long enough to capture this arrest, it is clear from the experiment using two-year-old trout that between 15 and 20 weeks after spawning, the control-ration fish began to increasingly partition energy to the ovary for developing oocytes.

At intake, three-year-old trout exhibited plasma E2 levels that were approximately an order of magnitude greater than those observed in two-year-old trout (Fig. 4), while ovarian masses were similar between the two age classes [42]. This difference in circulating E2 levels may be due to differences in the duration of pre-spawn fasting used by the facility from which trout were obtained (as described in Methods section), or to altered clearance of steroids by the liver [56], [57]. Previous work suggests that animals may experience an aging-associated decrease in the rate of steroid clearance via liver conjugation and hydroxylation reactions [58], [59], or an increasing sequestration of steroids by sex hormone-binding globulin [60], or some diffuse age-related effects associated with a diminished basal metabolic rate similar to that described in humans [61], [62] and other animals [63]. Regardless, this difference between age classes was transient: two-year-old and three-year-old fish exhibited similar plasma E2 levels by the second sampling date.

Our previous study [42] described how this feeding regime induced remodeling of organs and a general redistribution of energy stores, with control-ration fish accumulating lipid in muscle tissue and increasing liver mass. Interestingly, differences in plasma E2 between the two treatment groups were already significant at week ten and presumably were present sometime between weeks five and ten of the experiment. This would indicate that a trajectory of reproductive rematuration for the following year’s spawning effort had been at least partially determined approximately six weeks after spawning (as fish were obtained approximately one week after spawning), similar to the current understanding of the progression of puberty in salmonid fishes [29]. This implies that the energy deficit incurred during spawning and feeding conditions during the period immediately after spawning interact to determine whether rematuration is initiated within an approximately six week window. Restricted ration fish appear to have arrested rematuration before significant energy was invested in ovarian development.

Classic reproductive endocrine axis theory [2], [4] predicts that an increase in secretion of pituitary (*i.e.*, FSH) factors should precede an increase in plasma E2. However, among control ration fish, we detected no elevation in pituitary *fsh-β* mRNA (Fig. 5) expression prior to elevation of plasma E2 levels (Fig. 4). *In vitro* work suggests that FSH is regulated at the level of both transcription and secretion by the interaction of GnRH hierarchical stimulation and E2 feedback [64], and thus, pituitary expression of *fsh-β* may not be directly correlated with circulating levels of FSH peptide [65]. In addition, our ability to detect treatment differences in pituitary *fsh-β* mRNA was less than for plasma E2 due to the number of lethal and non-lethal samples at each time point (*e.g.*, for two-year-old trout at week 10, *n* = 60 for plasma E2, while *n* = 12 for pituitary *fsh-β* mRNA). Interestingly, within two-year-old trout, pituitary *fsh-β* mRNA expression was significantly higher among restricted-ration fish than control-ration fish between weeks 10 and 15; plasma E2 levels show the opposite trend and were elevated among control-ration fish during this time. This combination of observations is explained by the well-documented inhibitory tone of sex steroids on pituitary gonadotropin synthesis [66], [67], [68]. Among control-ration fish, increasing levels of plasma E2 exert negative feedback inhibition of *fsh-β* expression, as has been described in rainbow trout [69] and other fishes [70], [71]. Conversely, the lower plasma E2 levels observed among restricted-ration fish around this time releases pituitary *fsh-β* mRNA transcription from inhibition, causing an apparent increase in pituitary *fsh-β* mRNA levels among restricted-ration fish.

In a recent *in vitro* study in coho salmon, 11-KT stimulated growth of late perinucleolar stage follicles, suggesting a role for this androgen in early ovarian development [41]. However, it is not clear whether circulating or local levels of 11-KT are physiologically relevant in this scenario. In the present study, plasma 11-KT levels dropped rapidly between zero and five weeks after spawning and did not differ between treatment groups until week 25, which was 15 weeks after plasma E2 diverged. Therefore, our results do not support the hypothesis that plasma 11-KT stimulates ovarian rematuration during the period immediately following spawning. However, it cannot be ruled out that local 11-KT (*i.e.*, 11-KT produced in the ovary) may be involved in stimulating early ovarian development immediately after spawning, in a paracrine fashion. Between 20 and 25 weeks after spawning, treatment groups within the two-year-old trout diverged, with control ration fish exhibiting 2.4-fold elevated plasma levels of 11-KT versus restricted-ration fish by 30 weeks. This elevation in plasma 11-KT coincided with a significant elevation in GSI among control ration fish [42], suggesting that plasma 11-KT among maturing female fish may be of ovarian origin, as has previously been proposed [72]. Both age classes of trout started the experiment at the first sampling point immediately after spawning with elevated plasma 11-KT (Fig. 6), supporting the proposed role for 11-KT in female salmonid spawning physiology and behavior [37], [73], [74].

In conclusion, by restricting food availability in a group of female rainbow trout, growth and energy partitioning were both affected, with restricted-ration fish generally existing in a catabolic state and control-ration fish existing in an anabolic state for the duration of the experiment. Furthermore, the treatments were sufficient to induce differences in gonadal recrudescence between the two treatment groups: while restricted-ration fish arrested ovarian growth, control ration fish accumulated ovarian tissue until the end of the experiment [42]. Differences in ovary size were preceded by differences in circulating levels of E2, which diverged between one and two months after the start of the experiment. Although pituitary secretion of FSH presumably drives the increase in ovarian steroidogenesis underlying elevated plasma E2, our results suggests that this purported increase in FSH is not regulated at the level of transcription during the months immediately after spawning. In addition, our study provides no evidence to support the notion that circulating 11-KT is involved in early maturation of recrudescing female rainbow trout. Finally, food-restriction and the subsequent difference in reproductive trajectory did not measurably affect plasma levels of nesfatin-1 or ghrelin, suggesting that circulating levels of neither peptide link metabolic status to reproduction in *O. mykiss*. Future work should focus on elucidating the different roles of endocrine and paracrine nesfatin-1, as well as clarifying differences in chronic and acute nesfatin-1 responses to nutrient availability.
